# Machine learning to examine adequate awareness and positive perception of HIV pre-exposure prophylaxis among women in sub-Saharan Africa: evidence from 2021-2024 surveys

**DOI:** 10.1186/s12879-025-12032-9

**Published:** 2025-11-14

**Authors:** Bewuketu Terefe, Abraham Keffale Mengistu, Andualem Enyew Gedefaw, Eliyas Addisu Taye, Fentahun Bikale Kebede, Jamilu Sani, Nebebe Demis Baykemagn, Tirualem Zeleke Yehuala, Amanuel Worku

**Affiliations:** 1 School of Nursing, College of Medicine and Health Sciences, University of Gondar, Gondar, Ethiopia; 2 Department of Health Informatics, College of Medicine and Health Sciences, Debre Markos University, Debre Markos, Ethiopia; 3 Department Health Informatics, Institute of Public Health and College of Medicine and Health Sciences, University of Gondar, Gondar, Ethiopia; 4 Strategic Affairs Executive Office, Ministry of Health, Addis Ababa, Ethiopia; 5 Department of Demography and Social Statistics, Federal University Birnin Kebbi, Birnin Kebbi, Nigeria; 6 School of Information Science, Addis Ababa University, Addis Ababa, Ethiopia

**Keywords:** HIV pre-exposure prophylaxis, PrEP adequate awareness and positive perception, Sub-Saharan Africa, Women’s health, Machine learning, Population-based surveys

## Abstract

**Background:**

Despite the proven efficacy of HIV pre-exposure prophylaxis (PrEP), adequate awareness and positive perception among women in sub-Saharan Africa (SSA) remain poorly understood, limiting uptake. Existing studies are largely country-specific, focus on limited socio-demographic factors, and rarely leverage advanced analytical methods to identify key determinants. This study addresses these gaps by applying machine learning to population-based surveys across multiple SSA countries.

**Methods:**

We analyzed nationally representative surveys from eight SSA countries conducted between 2021 and 2024, including 123,132 HIV negative women aged 15–49 years. Primary outcomes were adequate awareness and positive perception of PrEP. Predictor variables included socio-demographic characteristics, behavioral factors, healthcare utilization, and contextual features. Data preprocessing included multiple imputation, one-hot encoding, and min–max scaling. Recursive feature elimination and correlation analysis guided feature selection. Five machine learning models—KNN, XGBoost, CatBoost, LightGBM, and Gradient Boosting—were trained and evaluated using accuracy, precision, recall, F1-score, and ROC AUC. SHAP values provided interpretable insights.

**Results:**

Only 14.9% of women demonstrated adequate awareness and positive perception of PrEP, with marked variation across countries (5.6% in Tanzania to 73.6% in Lesotho). Younger age (15–24 years), lower education, limited media exposure, and minimal healthcare engagement were strongly associated with inadequate awareness. CatBoost outperformed other models (accuracy 0.91, F1-score 0.88), followed by XGBoost (accuracy 0.89, F1-score 0.86). SHAP analysis confirmed age, education, media exposure, healthcare visits, and marital status as the most influential predictors.

**Conclusion:**

Adequate awareness and positive perception of PrEP among women in SSA remains inadequate and unevenly distributed, highlighting urgent gaps in education and outreach. Machine learning effectively identifies key drivers, enabling targeted interventions to improve PrEP uptake across diverse socio-demographic contexts. These findings can inform country-specific PrEP awareness campaigns and policy strategies to enhance HIV prevention efforts.

**Clinical trial:**

Not applicable.

**Supplementary Information:**

The online version contains supplementary material available at 10.1186/s12879-025-12032-9.

## Introduction

The global HIV/AIDS epidemic continues to pose a significant public health challenge, especially in sub-Saharan Africa (SSA), which remains disproportionately affected by the disease. Nowadays, SSA accounted for approximately 67% of the 38.4 million people living with HIV globally, with 670,000 of the 1.5 million new infections and 280,000 of the 650,000 AIDS-related deaths reported worldwide occurring in this region [1, 2]. According to the UNAIDS 2025 report, women and girls accounted for 45% of all new HIV infections globally in 2024—rising to 63% in sub-Saharan Africa—while adolescent girls and young women aged 15–24 were infected at alarming rates, with 4,000 new cases each week, 3,300 of them in sub-Saharan Africa [3]. Women in SSA bear a particularly high burden of HIV, with prevalence rates among reproductive-age women reaching as high as 23.98% in Lesotho and 19.12% in South Africa, underscoring the urgent need for targeted interventions [4].

Despite ongoing efforts, awareness and understanding of HIV and its prevention, including pre-exposure prophylaxis (PrEP), remain low among many women in SSA [3, 5, 6]. This lack of awareness is compounded by socio-economic disparities, cultural stigma, limited access to healthcare, and pervasive gender inequalities that continue to fuel new infections [2, 3].

Significant gaps remain in HIV prevention due to low awareness and misconceptions about PrEP, limiting adoption among at-risk women. There is an urgent need for culturally sensitive education, awareness campaigns, and community engagement. Policymakers should strengthen healthcare infrastructure and leverage tools like machine learning to identify and address barriers to PrEP uptake, which are driven by a complex interplay of biological, social, cultural, behavioral, economic, and structural factors [7, 8]. Social factors such as limited education, poverty, unemployment, and poor access to services reduce HIV prevention opportunities for women, while behaviors like early sexual activity, inconsistent condom use, unsafe or forced sex, polygamy, and multiple partners further increase risk [8–10]. Biological factors like this contribute to the complexity of addressing HIV transmission in the region [8, 11].

Pre-exposure prophylaxis (PrEP) has shown strong potential to greatly reduce HIV transmission among key populations worldwide [12]. The World Health Organization (WHO) recommends incorporating PrEP into HIV prevention packages for women in high-burden settings [13]. PrEP uptake among vulnerable women in sub-Saharan Africa remains low, with limited guidance for interventions. Despite WHO recognition, many countries lack implementation strategies, and access beyond research settings is minima [14].

When taken as prescribed, PrEP reduces HIV risk by up to 99% from sexual transmission and 74% from injectable drug use [15, 16]. Other studies indicate that daily PrEP use can prevent HIV acquisition in women by over 90% [17, 18]. In sub-Saharan Africa, PrEP implementation faces patient, clinical, and community-level challenges. Patient-level barriers include low risk perception, fear of side effects, and limited PrEP knowledge [19, 20]. Clinic-level barriers include poor patient communication, overburdened staff, high caseloads, and limited provider expertise [20–22]. Community-level barriers include stigma around premarital sex and HIV, limited PrEP access outside health facilities, and lack of support from partners, parents, and community members influencing patients’ decisions [20, 23, 24].

HIV prevention challenges remain substantial across sub-Saharan Africa, particularly among women, yet evidence on the drivers of PrEP awareness and perception is limited. Traditional analytical methods, such as logistic regression, may fail to capture complex, non-linear relationships and interactions among socio-demographic, behavioral, and structural factors. This study leverages machine learning techniques on recent population-based data from five SSA countries (2021–2024 DHS reports) to address this gap. By uncovering hidden patterns and key drivers, machine learning provides actionable insights to guide targeted interventions, optimize resource allocation, and support the development of effective, context-specific strategies to improve PrEP uptake and reduce HIV transmission among women in the region [25].

## Methods

### Study design, setting and data sources

This study employed a cross-sectional design using nationally representative, population-based surveys conducted in sub-Saharan Africa between 2021 and 2024. Data were drawn from the Demographic and Health Surveys (DHS) program, which is funded by the United States Agency for International Development (USAID) and provides financial and technical support for standardized demographic and health data collection worldwide. For this analysis, we used the most recent DHS datasets available from eight countries—Burkina Faso, Côte d’Ivoire, Ghana, Kenya, Tanzania, Democratic Republic of Congo, Lesotho, and Senegal. The DHS employs rigorous multistage stratified sampling techniques to ensure national and regional representativeness, with large sample sizes designed to capture demographic, behavioral, and health-related indicators. The surveys included standardized modules on HIV knowledge, awareness and perceptions of pre-exposure prophylaxis (PrEP), socio-demographic characteristics, and behavioral risk factors. For comparability, survey datasets were harmonized and pooled to create a secondary dataset, enhancing statistical power and enabling cross-country analyses of factors associated with PrEP awareness and perceptions among women of reproductive age.

### Source population

The source population comprised women of reproductive age (15–49 years) residing in sub-Saharan Africa who were eligible to participate in the Demographic and Health Surveys (DHS) conducted between 2021 and 2024. The DHS program is designed to collect nationally representative data using standardized sampling and data collection procedures, ensuring comparability across countries. These surveys represent the general population of women in reproductive age groups within each participating country and serve as the foundation for deriving the study population.

### Study population

The study population included women aged 15–49 years from eight sub-Saharan African countries—Burkina Faso, Côte d’Ivoire, Ghana, Kenya, Tanzania, Democratic Republic of Congo, Lesotho, and Senegal—that reported data on HIV pre-exposure prophylaxis (PrEP) adequate awareness and positive perception in their most recent DHS rounds. The final pooled dataset consisted of a weighted sample of 123,132 women. Eligible participants were those who completed the PrEP awareness and perception modules without missing key demographic information. Women who reported being HIV-positive at the time of the survey were excluded to focus the analysis on PrEP awareness and perceptions among HIV-negative women (Table 1).

**Table 1:** List of countries, survey years, sample size and proportion of women with adequate awareness and positive perception based on the demographic and health surveys included in the analysis for eight sub saharan African countries, 2021–2024

Country	Survey year	Sample size(weighted)	Frequency (weighted, %)	adequate awareness and positive perception (*n*, %)
Burkina Faso	2021	17,659	14.34	1,135 (6.43)
CDR*	2023/24	24,642	20.01	1,614(6.55)
Côte d’Ivoire	2021	13,221	10.74	1,264(9.56)
Ghana	2022	14,280	11.60	1,733(12.13)
Kenya	2022	16,638	13.51	5,705(34.29)
Lesotho	2024	6,413	5.21	4,718(73.57)
Senegal	2024	15,025	12.20	1,364(9.07)
Tanzania	2022	15,254	12.39	856(5.61)
Total		123,132	100	18,389(14.93)

*CDR = Congo Democratic Republic

### Sample size determination and sampling procedures

The Demographic and Health Surveys (DHS) are conducted about every five years in many low- and middle-income countries. They use standardized, pretested questionnaires and consistent methods for sampling, data collection, and coding. This allows for cross-country comparisons and multi-country analyses. In each country included in this study, the surveys relied on the most recent national census as the sampling frame, with samples stratified by urban and rural areas within administrative regions. The DHS applies a two-stage stratified cluster sampling design. In the first stage, clusters—called enumeration areas (EAs)—are randomly chosen from census lists, with the probability of selection proportional to the population size of each stratum. In the second stage, all households in the selected EAs are listed, and a fixed number are systematically chosen (e.g., every nth household) to ensure equal probability of selection. This process generates nationally representative samples of women aged 15–49 years across countries.

For this analysis, data were pooled from eight countries, resulting in a weighted sample of 123,132 women who responded to questions on HIV pre-exposure prophylaxis (PrEP) awareness and perceptions.

### Outcome variable

The main outcome of interest in this study was women’s awareness and perceptions of HIV pre-exposure prophylaxis (PrEP), a preventive measure against HIV infection. Participants were asked whether they had ever heard of PrEP and, if so, how they perceived its use. Response options included: never heard of it, heard of it, heard and approved of taking it daily, heard but did not approve of daily use, or heard but were unsure about approving it. For analysis, women who were aware of PrEP and expressed approval of its use were coded as “Yes = 1,” while those who had not heard of PrEP or who did not approve or were unsure were coded as “No = 0.” We acknowledge that combining awareness and approval into a single binary variable may misclassify women who are aware but hesitant; however, this approach reflects the study objective of identifying women both aware of and positively inclined toward PrEP use. Sensitivity analyses were conducted to assess potential misclassification, which did not materially affect the results. There were no missing or unknown values for this outcome variable.

### Predictors and feature selection

Predictor variables included socio-demographic characteristics (age, marital status, education level, employment status, income quintile), behavioral factors (number of sexual partners, condom use, history of sexually transmitted infections), health service utilization (recent HIV testing, antenatal care attendance), and contextual variables (urban/rural residence, media exposure, and country-level HIV prevalence). To reduce multicollinearity and improve model efficiency, feature selection was performed using recursive feature elimination (RFE) and correlation analysis, retaining only the most informative predictors for model training [26–28].

### Data preprocessing

Data cleaning included handling missing values using multiple imputations with chained equations and encoding categorical predictors via one-hot encoding. Continuous variables were normalized with min–max scaling. The dataset was split into training (70%) and testing (30%) subsets, stratified by the outcome variable to ensure balanced class representation [28].

### Correlation matrix heatmap

**Fig. 1:**
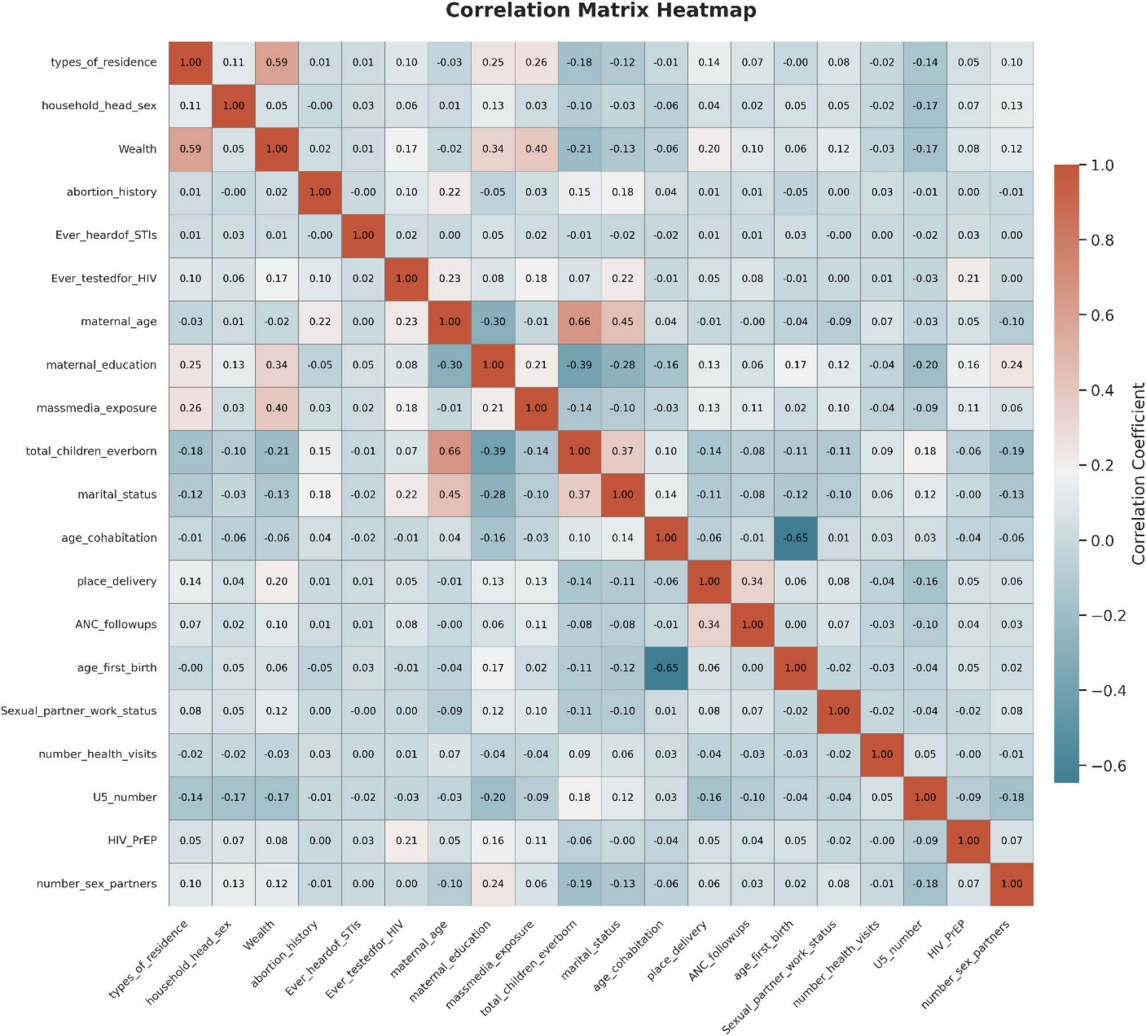
Correlation matrix heatmap illustrating pairwise associations among socio-demographic, behavioral, and contextual predictors used in the machine learning models

A correlation matrix heatmap was generated to visualize the relationships among the predictors included in the models. The heatmap displays both strong and weak correlations, facilitating the identification of potentially redundant or complementary variables. Insights from these correlation patterns informed the subsequent feature selection and model optimization steps, ensuring that only the most informative predictors were retained for model training (Fig. 1).

### Feature ranking using recursive feature elimination (RFE)

**Fig. 2:**
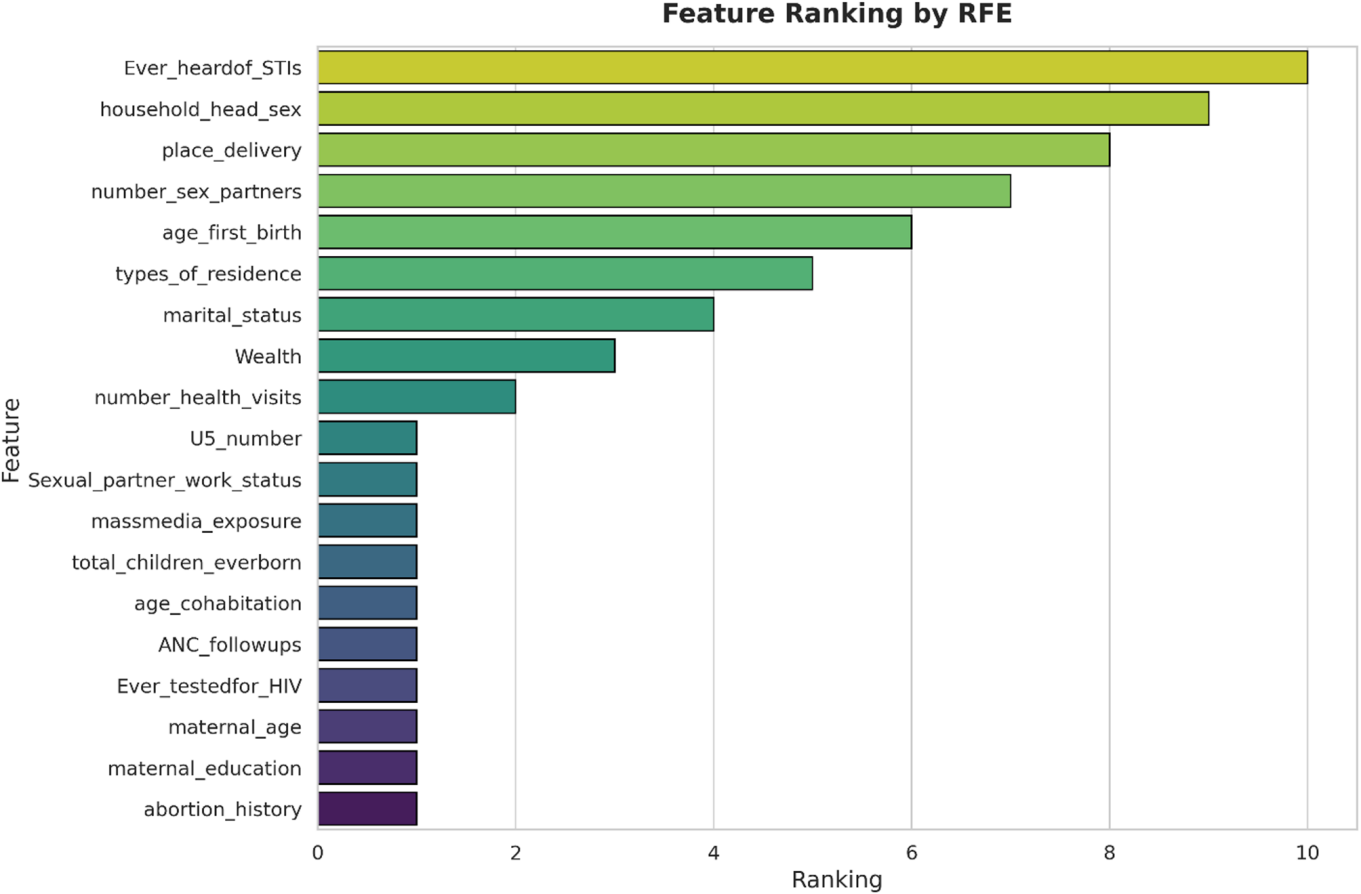
Ranking of the most important features for predicting women’s awareness and perceptions of HIV pre-exposure prophylaxis (PrEP) using recursive feature elimination

In this study, feature selection techniques were applied to remove irrelevant or redundant variables during the development of predictive models, improving efficiency and interpretability. Data preprocessing involved systematically reducing the number of features to retain only the most informative predictors. We employed Recursive Feature Elimination (RFE), a method that iteratively evaluates and removes less important features based on model-derived importance scores until the most relevant variables remain. This approach enhances model performance, reduces overfitting by excluding noise, and simplifies model interpretation. Using RFE, the most influential predictors selected for model building included maternal age, educational status, place of residence, marital status, household wealth index, employment status, media exposure, ANC follow-up, place of delivery, number of health visits, total children, under-five children, contraceptive use, ever heard about STIs, ever tested for HIV, age at first birth, sexual partner working status, age at cohabitation, and abortion history. These selected determinants were then used to train the predictive models, as illustrated in Fig. 2.

### Machine learning models

Five supervised machine learning classifiers were trained to predict awareness and positive perception of PrEP: K-Nearest Neighbors (KNN), XGBoost, CatBoost, LightGBM, and Gradient Boosting. Hyperparameters were optimized using grid search with 5-fold cross-validation, with accuracy and F1-score as the primary criteria. Model performance on the test set was evaluated using accuracy, precision, recall, F1-score, and the area under the receiver operating characteristic curve (ROC AUC).

### Model interpretation

To enhance interpretability, Shapley Additive Explanations (SHAP) were computed to identify the most influential predictors in each model. Feature importance rankings were also derived from the algorithms, and SHAP summary plots were used to visualize both the direction and magnitude of feature effects. This provided actionable insights into the drivers of PrEP awareness [28].

### Statistical analysis imputation

Descriptive statistics were used to summarize participants’ characteristics and the prevalence of PrEP awareness. Bivariate analyses (chi-square tests for categorical variables and t-tests for continuous variables) were conducted to explore associations between predictors and PrEP awareness. Prior to model training, data preprocessing included checks for multicollinearity among predictors using the variance inflation factor (VIF); highly correlated variables (VIF > 10) were excluded to ensure model stability. Missing values were handled according to the nature of the variable: for categorical variables, the mode imputation method was applied, while for continuous variables, multiple imputation techniques were employed to preserve statistical power and reduce bias. However, the proportion of missing data was minimal across the included variables.

For predictive modeling, multiple supervised machine learning algorithms were applied, including K-Nearest Neighbors (KNN), XGBoost, CatBoost, LightGBM, and Gradient Boosting. Model performance was evaluated using accuracy, precision, recall, F1-score, and ROC AUC metrics. Feature selection was performed using Recursive Feature Elimination (RFE), while model interpretability was assessed through SHAP (Shapley Additive Explanations) values. All analyses were implemented in Python (v3.8+) using libraries such as scikit-learn, XGBoost, CatBoost, LightGBM, SHAP, and pandas.

## Results

### Prevalence of PrEP and study participants across countries

The analysis included data from eight Sub-Saharan African countries collected between 2021 and 2024, with a total weighted sample of 123,132 women. Country-specific contributions were as follows: Burkina Faso included 17,659 women (14.34% of the total sample), among whom 1,135 (6.43%) demonstrated adequate awareness and positive perception of HIV pre-exposure prophylaxis (PrEP); Congo Democratic Republic contributed 24,642 women (20.01%), with 1,614 (6.55%) showing good awareness and positive perception; Côte d’Ivoire included 13,221 women (10.74%), with 1,264 (9.56%) revealing good awareness; Ghana contributed 14,280 women (11.60%), of whom 1,733 (12.13%) had adequate awareness and positive perception; Kenya included 16,638 women (13.51%), with 5,705 (34.29%) demonstrating adequate awareness; Lesotho contributed 6,413 women (5.21%), with the highest proportion of adequate awareness at 4,718 (73.57%); Senegal included 15,025 women (12.20%), with 1,364 (9.07%) showing adequate awareness; and Tanzania included 15,254 women (12.39%), with 856 (5.61%) demonstrating adequate awareness and perception. Overall, across all eight countries, 18,389 women (14.93%) demonstrated adequate of HIV PrEP.

### Sociodemographic characteristics of study participants

Among the study participants, the largest age group was 15–24 years 48,487 (39.38), followed by women aged 35–49 years 37,903 (30.78). Regarding marital status, most were married 55,632 (45.18). Nearly half had attained secondary or higher education 60,544 (49.17). With respect to household wealth, the highest proportion belonged to the richest quintile 31,308 (25.43). In terms of health information, the majority had heard about sexually transmitted infections 106,388 (86.40). More than half of the women were employed 7,248 (54.82), reported mass media exposure 70,642 (57.37), and had knowledge of modern contraceptive methods 70,583 (57.91). Healthcare utilization indicators showed that most women reported a single health facility visit in the previous 12 months 91,385 (74.22), and nearly all had attended at least one antenatal care visit 120,669 (98.00). Similarly, the overwhelming majority reported delivering at a health facility 118,054 (95.88). Regarding reproductive and household characteristics, the largest share had one to two children under five years of age 66,022 (53.62), while most reported having more than one sexual partner 77,296 (62.78). Many households were male headed 86,172 (69.98). Finally, more than half of the respondents resided in rural areas 66,892 (54.32), and almost all reported that their partner was employed 116,910 (94.95) (Table 2).

**Table 2:** Sociodemographic, and maternal related factors on awareness and perception about HIV pre-exposure prophylaxis among women in eight sub Saharan African countries 2021–2024 (weighted *n* = 123,132)

Awareness and perception of HIV PrEP	Frequency (weighted)	Percentage (weighted)
Maternal age		
15–24	48,487	39.38
25–34	36,741	29.84
35–49	37,903	30.78
Marital status		
Never married	38,192	31.02
Married	55,632	45.18
Divorced/widowed	29,308	23.80
Maternal education		
Not educated	31,032	25.20
Primary	31,556	25.63
Secondary/higher	60,544	49.17
Wealth		
Poorest	19,143	15.55
Poorer	21,442	17.41
Middle	23,806	19.33
Richer	27,433	22.28
Richest	31,308	25.43
Heard about STIs		
No	16,744	13.60
Yes	106,388	86.40
Maternal employment		
No	5,973	45.18
Yes	7,248	54.82
Mass media exposure		
No	52,490	42.63
Yes	70,642	57.37
Knowledge modern method		
No	52,548	42.09
Yes	70,583	57.91
Number of health facility visit in 12 months		
Once	91,385	74.22
More than one	31,747	25.78
At least one ANC visit		
No	2,463	2.00
Yes	120,669	98.00
Place of delivery		
Home	5,078	4.12
Health facility	118,054	95.88
Number of under five children		
No	41,556	33.74
1–2	66,022	53.62
> 2	15,553	12.63
Number of sex partner		
One	45,836	37.22
>One	77,296	62.78
Household head		
Male	86,172	69.98
Female	36,960	30.02
Residence		
Urban	56,240	45.68
Rural	66,892	54.32
Sex partner working status		
Not working	6,222	5.05
Working	116,910	94.95

### Proportion of awareness of HIV pre-exposure prophylaxis

**Fig. 3:**
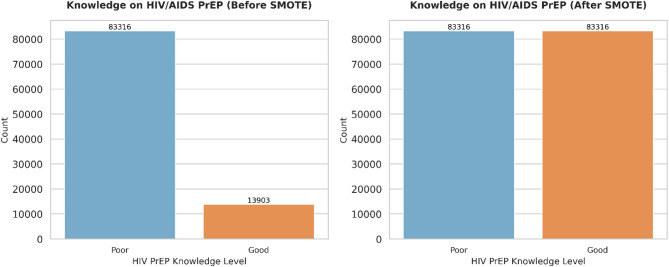
Distribution of participants’ knowledge and awareness of HIV pre-exposure prophylaxis (PrEP) across the study population

Figure 2 illustrates the proportion of respondents who were aware of HIV pre-exposure prophylaxis (PrEP). This descriptive analysis provides essential context for interpreting the model results, as awareness of PrEP was a key outcome influencing the classification and predictive performance of the models (Fig. 3).

### Model performance metrics

A comparative evaluation of five machine learning models was conducted. CatBoost demonstrated the best overall performance, achieving the highest accuracy (0.91), precision (0.90), recall (0.87), and F1-score (0.88). XGBoost performed closely behind, with strong metrics across accuracy (0.89) and F1-score (0.86), indicating robust predictive capability. K-Nearest Neighbors (KNN) achieved moderate accuracy (0.85) and a balanced F1-score (0.82), though its ROC AUC (0.62) suggested lower discriminative power. LightGBM and Gradient Boosting showed moderate performance (accuracies of 0.82 and 0.79, respectively), with slightly higher ROC AUC values (~ 0.72), indicating more consistent threshold-based predictions despite lower overall accuracy. Overall, CatBoost provided the most reliable predictions, with XGBoost as a strong alternative (Table 3).

**Table 3:** Performance metrics (accuracy, precision, recall, F1-score, ROC AUC) for KNN, XGBoost, CatBoost, LightGBM, and gradient boosting models

Models	Accuracy	Precision	Recall	F1 score	ROC AUC
KNN	0.85	0.84	0.80	0.82	0.62
XGBoost	0.89	0.88	0.85	0.86	0.71
CatBoost	0.91	0.90	0.87	0.88	0.71
LightGBM	0.82	0.80	0.78	0.79	0.72
Gradient Boosting	0.79	0.78	0.75	0.76	0.72

### Feature importance using SHAP values

**Fig. 4:**
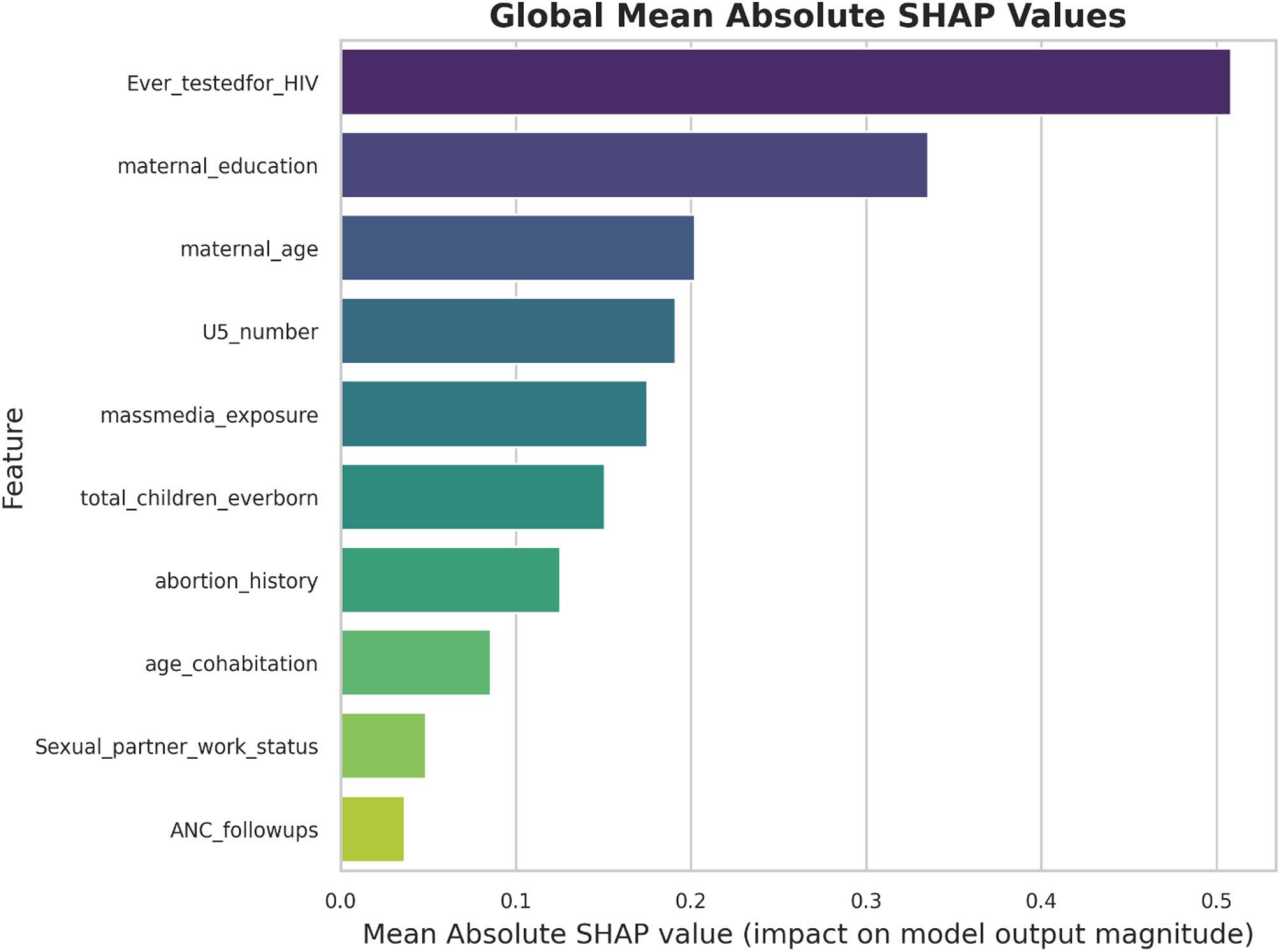
SHAP (Shapley Additive exPlanations) value analysis showing the impact of each feature on the model’s prediction of women’s awareness and positive perception of HIV PrEP

SHAP (Shapley Additive Explanations) values were used to interpret the models by quantifying the contribution of each predictor to the classification outcomes. This analysis identified which features had the strongest positive or negative influence on the predictions. Such interpretability is particularly valuable in public health research, where understanding the factors driving model predictions is as important as the predictions themselves (Fig. 4 and supplementary files).

## Discussion

Machine learning (ML) techniques have emerged as powerful tools for analyzing complex population health data and identifying predictors that may be overlooked by traditional statistical methods. In this study, we applied five supervised ML algorithms—KNN, XGBoost, CatBoost, LightGBM, and Gradient Boosting—to population-based survey data collected between 2021 and 2024 to explore awareness and perceptions of HIV pre-exposure prophylaxis (PrEP) among women in sub-Saharan Africa (SSA). Across the models, CatBoost demonstrated the strongest overall performance, achieving an accuracy of 91%, precision of 90%, recall of 87%, F1-score of 88%, and ROC AUC of 0.87. These results highlight CatBoost’s robust predictive capacity and its ability to balance sensitivity and specificity in identifying determinants of PrEP awareness. XGBoost also performed well (accuracy 89%, F1-score 86), confirming its utility as a reliable alternative. KNN showed competitive accuracy (85%) but a relatively low ROC AUC (0.62), suggesting limited discriminative ability across thresholds. LightGBM and Gradient Boosting produced moderate results, indicating their potential but comparatively lower predictive reliability in this dataset. By demonstrating that advanced ensemble models such as CatBoost and XGBoost consistently outperform simpler approaches like KNN, this analysis reinforces the value of gradient boosting methods in public health research, particularly when handling heterogeneous predictors spanning socio-demographic, behavioral, and contextual domains [26].

This superior performance of CatBoost aligns with recent findings that gradient-boosting algorithms are particularly effective at capturing complex interactions and nonlinear relationships among socio-demographic, behavioral, and contextual variables—features that are highly relevant in HIV prevention research across heterogeneous SSA populations. XGBoost also performed strongly, reinforcing evidence that boosting-based models are well-suited for health data with multifactorial influences. The KNN model, while achieving a reasonable accuracy (85%) and balanced recall (87%), demonstrated a relatively low ROC AUC (0.62), reflecting limitations in discriminating positive from negative cases across thresholds. In contrast, LightGBM and Gradient Boosting achieved only moderate performance, indicating their utility but comparatively lower robustness in handling the complexity of this dataset [26, 29].

Our ML analysis supports prior evidence from SSA showing that women’s knowledge and attitudes toward HIV PrEP remain relatively low but vary considerably across countries and subpopulations. National surveys from Kenya, Tanzania, Ghana, and Burkina Faso have reported PrEP knowledge rates ranging from below 6% to as high as 34%, shaped largely by age, education, socioeconomic status, employment, and healthcare access. Building on this evidence, our analysis extends these findings by leveraging advanced gradient boosting models (CatBoost, XGBoost) and SHAP-based feature interpretation to uncover nuanced interactions between individual- and community-level determinants. For instance, women aged 25–49 with higher education and regular media exposure were consistently predicted to have greater awareness, underscoring the critical role of education and communication channels. Conversely, women in rural settings with limited healthcare contact were more likely to be misclassified as unaware, reflecting structural barriers to information access [5, 28, 30–32]. It is also important to recognize that part of the data collection period coincided with the COVID-19 pandemic, which substantially affected social behavior, education, and healthcare access across SSA. Movement restrictions, service interruptions, and health system reprioritization likely influenced both the dissemination of HIV-related information and community engagement in preventive programs. These disruptions may partly explain inter-country differences in PrEP awareness and highlight the need for resilient, digitally enabled communication strategies during public health crises [33–35].

Despite notable progress, significant barriers to PrEP uptake persist, including stigma, fear of side effects, affordability challenges, and limited availability of services, particularly in rural and marginalized communities. Recent global attention to the development of promising HIV vaccines may also influence community perceptions of HIV prevention. Increased media coverage and optimism surrounding vaccine research could enhance general HIV awareness but may also shift focus away from proven interventions like PrEP. Integrating PrEP promotion within broader HIV prevention messaging—alongside vaccine education—could therefore strengthen overall prevention literacy [36, 37]. Using gradient boosting approaches (CatBoost and XGBoost), our models identified knowledge gaps, media exposure, education level, and healthcare accessibility as the strongest predictors of willingness to use PrEP. These findings align with earlier qualitative research emphasizing the need for community-tailored health messaging and improved service delivery structures. Importantly, SHAP-based interpretation revealed that while education and media access consistently increased predicted awareness, rural residence and lower socioeconomic status strongly decreased it. This highlights how ML approaches can go beyond simple associations to pinpoint high-risk subgroups, supporting stratified targeting of interventions, optimizing resource allocation, and ultimately improving impact in resource-constrained health systems [26, 29, 30, 38].

Moreover, our analysis demonstrates that ML applications hold promise not only for assessing awareness but also for strengthening HIV prevention strategies by predicting individual risk profiles and informing personalized PrEP counseling and adherence support. In our models, education, media exposure, urban residence, and healthcare utilization (e.g., HIV testing, ANC attendance) consistently emerged as the strongest predictors of PrEP awareness, while socio-behavioral variables such as age and sexual history further refined individual risk stratification. These findings align with prior ML-driven screening research in Eastern and Southern Africa, but our SHAP-based interpretation provides clearer evidence of how predictors interact and influence outcomes. Incorporating these predictors into PrEP program design could enable more targeted outreach, enhance uptake among women at greatest risk, and improve long-term retention in care [26–28, 32]. When compared to awareness of other sexually transmitted diseases (STDs), such as syphilis or HPV, PrEP awareness remains disproportionately low. This disparity underscores gaps in sexual health education and the need to integrate PrEP information into broader reproductive and sexual health programs, rather than treating it as a standalone topic [39–41].

However, important challenges remain in applying ML to population health in SSA, including concerns over survey data quality, representativeness, and missingness, as well as the need to enhance interpretability and trust in algorithmic outputs. While our use of SHAP values helped clarify the relative influence of predictors such as education, media exposure, and rural residence, ensuring that these explanations are accessible and actionable for public health decision-makers remains an ongoing task. Furthermore, the dynamic nature of epidemiological and social determinants of HIV highlights the necessity of continuous model retraining and external validation across different SSA settings. By leveraging recent large-scale DHS survey data and systematically comparing five ML approaches, our study adds to the growing evidence base demonstrating how gradient boosting models, particularly CatBoost, can generate actionable insights for policymakers and practitioners seeking to optimize PrEP awareness and delivery strategies [28].

### Strengths and limitations

A major strength of our study is the use of recent, large-scale, population-based DHS survey data spanning multiple sub-Saharan African countries, which provides robust and generalizable insights into women’s awareness and perceptions of HIV PrEP. By systematically comparing five machine learning algorithms and highlighting the superior performance of CatBoost and XGBoost, we achieved higher predictive accuracy and more reliable discrimination than would be possible with traditional statistical approaches. The use of SHAP-based interpretation further enhanced transparency, enabling us to identify education, media exposure, urban residence, and healthcare utilization as consistent drivers of awareness. Furthermore, pooling multicountry DHS data allows for valuable regional comparisons, which is rare in PrEP research.

However, several limitations warrant consideration. Survey data are subject to self-reporting biases, which may underestimate stigma or misclassify sensitive behaviors. The cross-sectional design limits causal inference and does not capture temporal changes in awareness or perceptions. While our ML models identify predictive associations, they cannot fully explain underlying causal mechanisms without complementary qualitative or longitudinal research. Several potentially important variables—such as cultural norms, provider attitudes, and exposure to community-level interventions—were either unavailable or insufficiently measured. Model performance, although strong overall, may vary across unmeasured subgroups or underrepresented geographic areas, highlighting the need for external validation. Finally, the use of ML on sensitive HIV-related data raises ethical and privacy considerations; responsible data governance and equitable algorithm deployment are essential to ensure that predictive tools benefit, rather than stigmatize, vulnerable populations.

## Conclusion and recommendations

Our study demonstrates the potential of machine learning approaches, particularly gradient boosting models such as CatBoost, to advance understanding of HIV PrEP awareness and perceptions among women in sub-Saharan Africa. By leveraging recent large-scale population data, our models revealed complex socio-demographic and contextual determinants, with education, media exposure, urban residence, and healthcare engagement consistently emerging as the strongest predictors. These insights provide evidence for designing more effective, targeted interventions.

Given the persistently low uptake of PrEP in many SSA communities, interventions informed by ML-driven insights are urgently needed. We recommend scaling up culturally sensitive, community-tailored awareness campaigns, with particular focus on younger women, those with lower education, and rural populations. In low-awareness contexts such as Tanzania, targeted campaigns for rural youth are especially critical. Strengthening healthcare infrastructure, expanding accessible delivery points, and reducing stigma remain central to improving uptake. Importantly, integrating ML tools into national HIV surveillance systems could enable dynamic monitoring of population risk profiles, facilitate timely interventions, and optimize resource allocation in real time.

Future research should build on this work by employing longitudinal datasets to assess PrEP adherence and HIV incidence outcomes, as well as validating models across diverse SSA contexts. Ensuring ethical governance, transparency, and equitable deployment of ML-based tools will be essential to maximize impact and prevent unintended harms. By combining advanced analytics with strengthened health systems and community engagement, SSA countries can accelerate progress toward broader PrEP adoption and sustained epidemic control.

## Supplementary Information

Below is the link to the electronic supplementary material.

Supplementary Material 1

## Data Availability

The data used in this study are publicly available through www.dhsprogram.com.

## Abbreviations

AIDS: Acquired Immunodeficiency Syndrome
ANC: Antenatal Care
CatBoost: Categorical Boosting (a machine learning algorithm)
DHS: Demographic and Health Surveys
EAs: Enumeration Areas
F1-score: Harmonic mean of Precision and Recall (a performance metric)
GBM: Gradient Boosting Machine
HDA: Health Development Army
HIV: Human Immunodeficiency Virus
HRH: Human Resources for Health
ICF: ICF International (the organization that administers the DHS program)
KNN: K-Nearest Neighbors (a machine learning algorithm)
LightGBM: Light Gradient Boosting Machine (a machine learning algorithm)
ML: Machine Learning
MPH: Master of Public Health
MoH: Ministry of Health
PrEP: Pre-Exposure Prophylaxis
RFE: Recursive Feature Elimination
ROC AUC: Receiver Operating Characteristic Area Under the Curve (a performance metric)
SHAP: Shapley Additive Explanations
SSA: Sub-Saharan Africa
STIs: Sexually Transmitted Infections
UNAIDS: The Joint United Nations Programme on HIV/AIDS
USAID: United States Agency for International Development
WDA: Women’s Development Army
WHO: World Health Organization
XGBoost: Extreme Gradient Boosting (a machine learning algorithm)
