# Supplementary material for: Machine learning to examine adequate awareness and positive perception of HIV pre-exposure prophylaxis among women in sub-Saharan Africa: evidence from 2021-2024 surveys
Source: BMC Infect Dis. 2025 Nov 14;25:1580. doi: 10.1186/s12879-025-12032-9 (PMC12619335; doi:10.1186/s12879-025-12032-9)
Supplement: Supplementary file 1 — Supplementary Material 1 [file 12879_2025_12032_MOESM1_ESM.docx]

Suplementary files


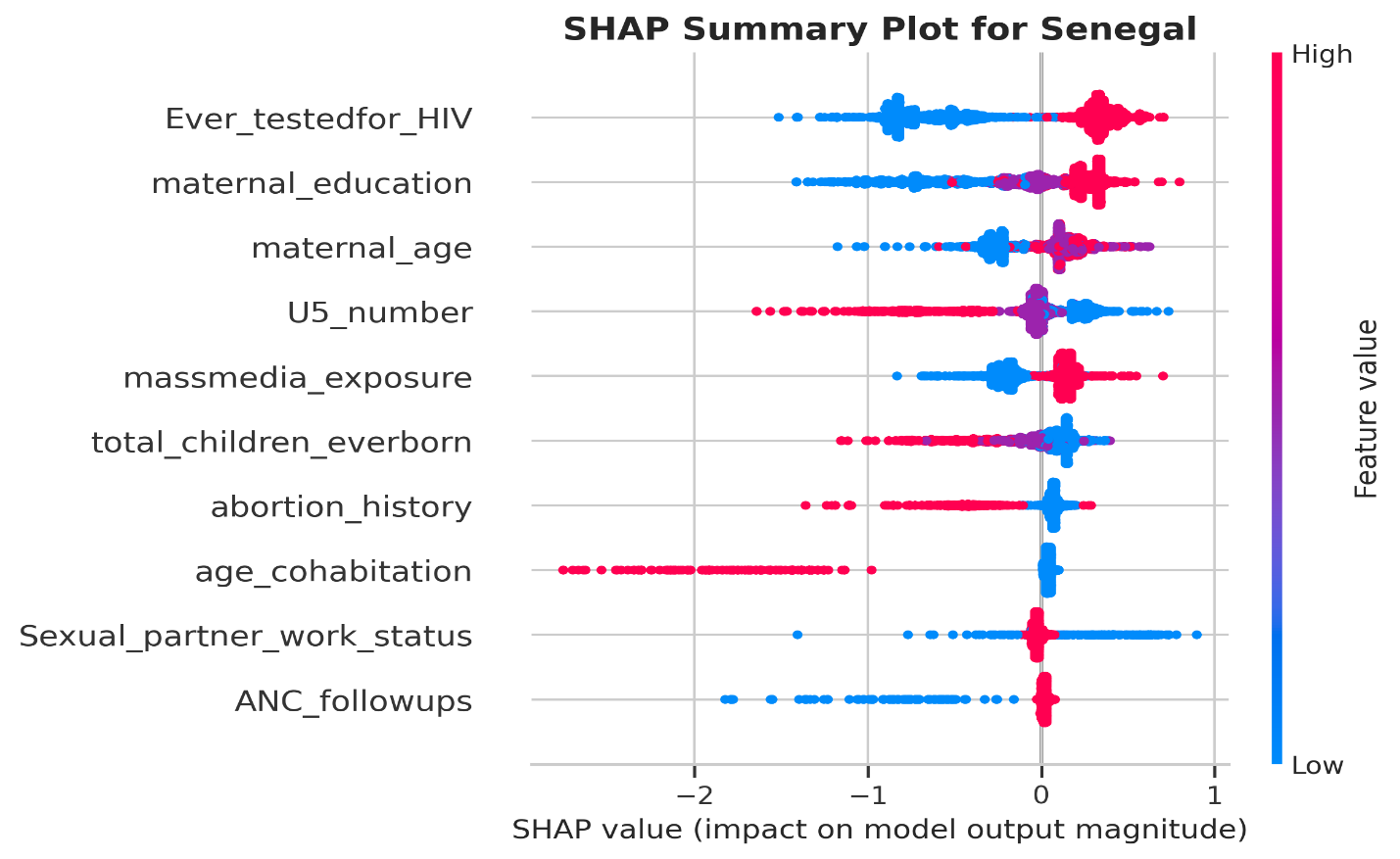


Supplementary Figure 1. SHAP (Shapley Additive exPlanations) summary plot illustrating the impact and direction of each predictor variable on the model’s prediction of women’s awareness and favorable perception of HIV pre-exposure prophylaxis (PrEP) in Senegal.


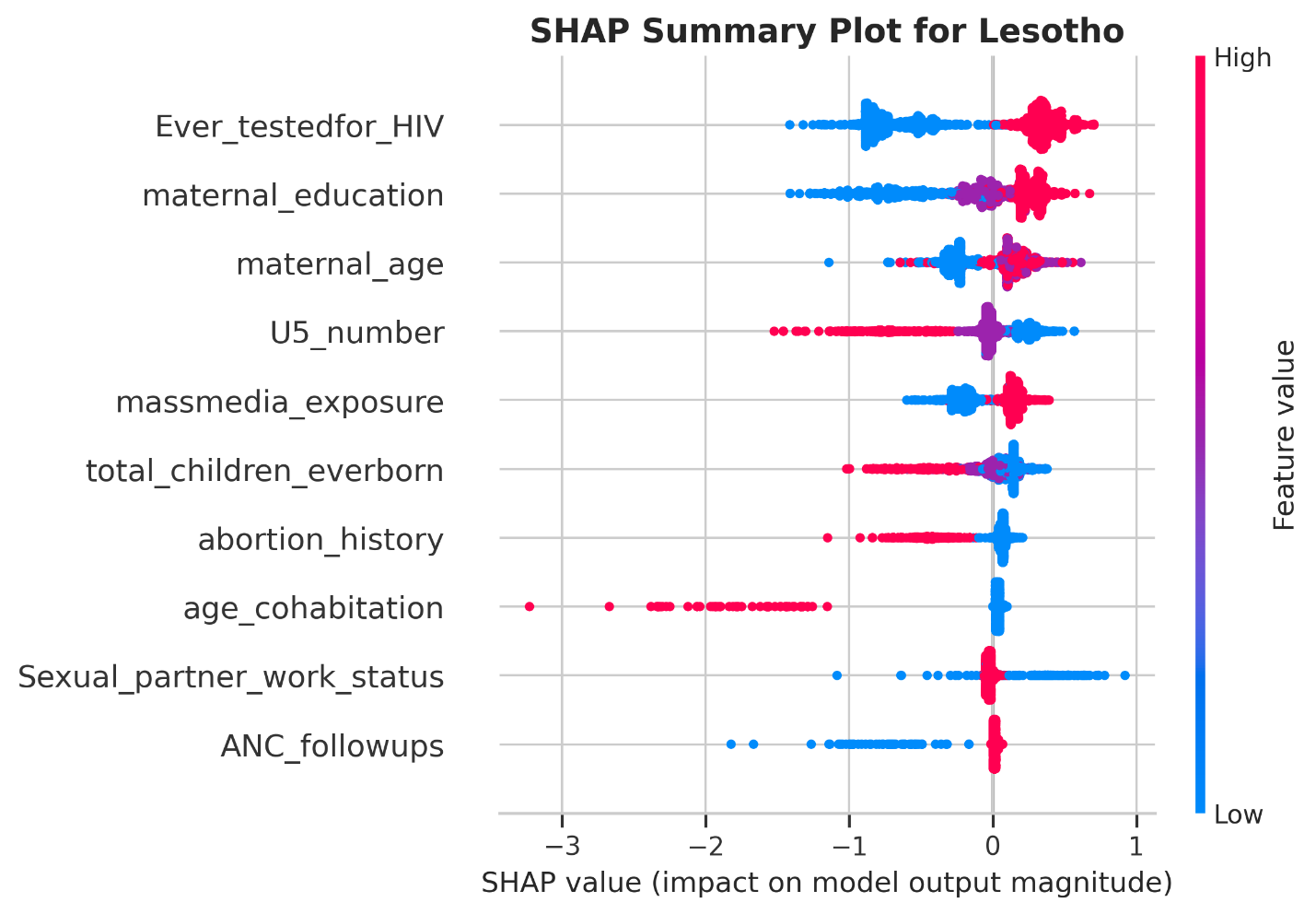


Supplementary Figure 2. SHAP (Shapley Additive exPlanations) summary plot illustrating the impact and direction of each predictor variable on the model’s prediction of women’s awareness and favorable perception of HIV pre-exposure prophylaxis (PrEP) in Lesotho.


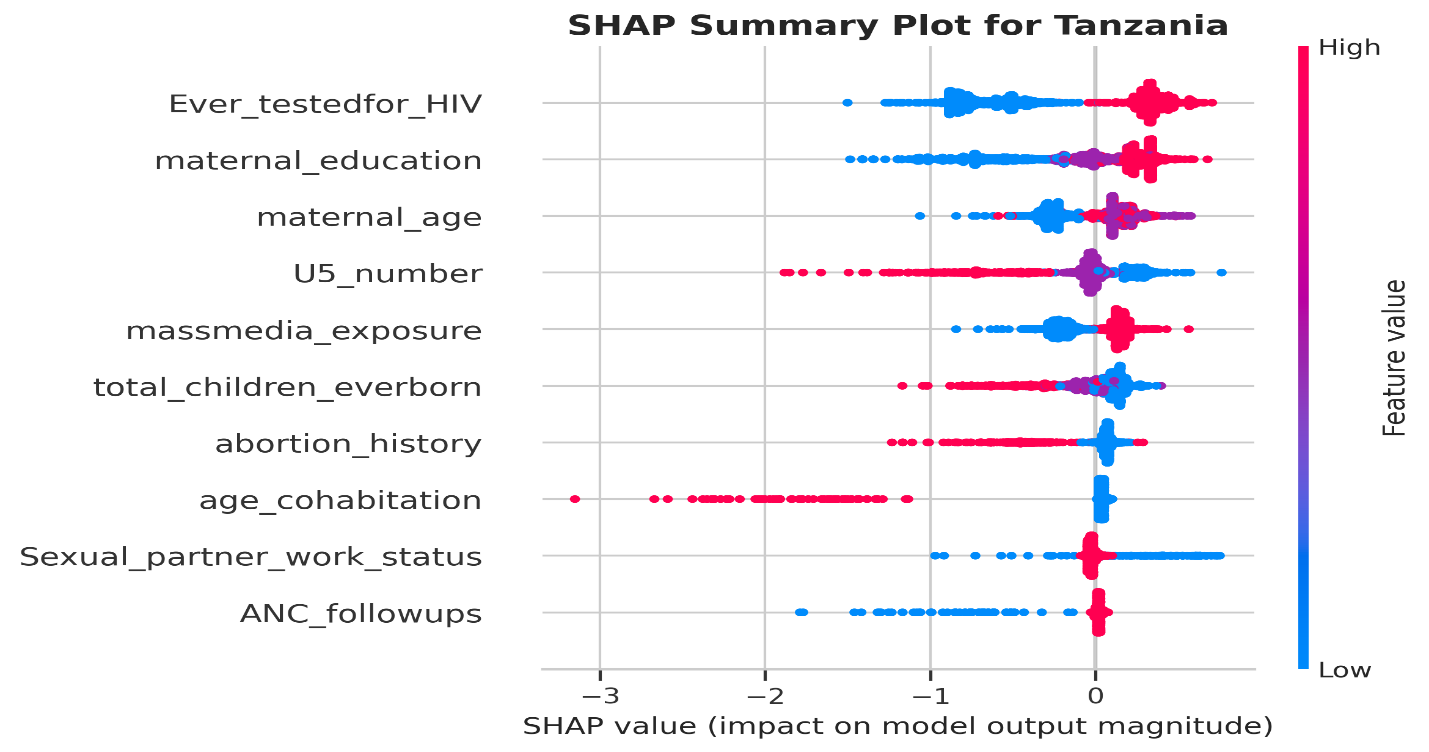


Supplementary Figure 3. SHAP (Shapley Additive exPlanations) summary plot illustrating the impact and direction of each predictor variable on the model’s prediction of women’s awareness and favorable perception of HIV pre-exposure prophylaxis (PrEP) in Tanzania.


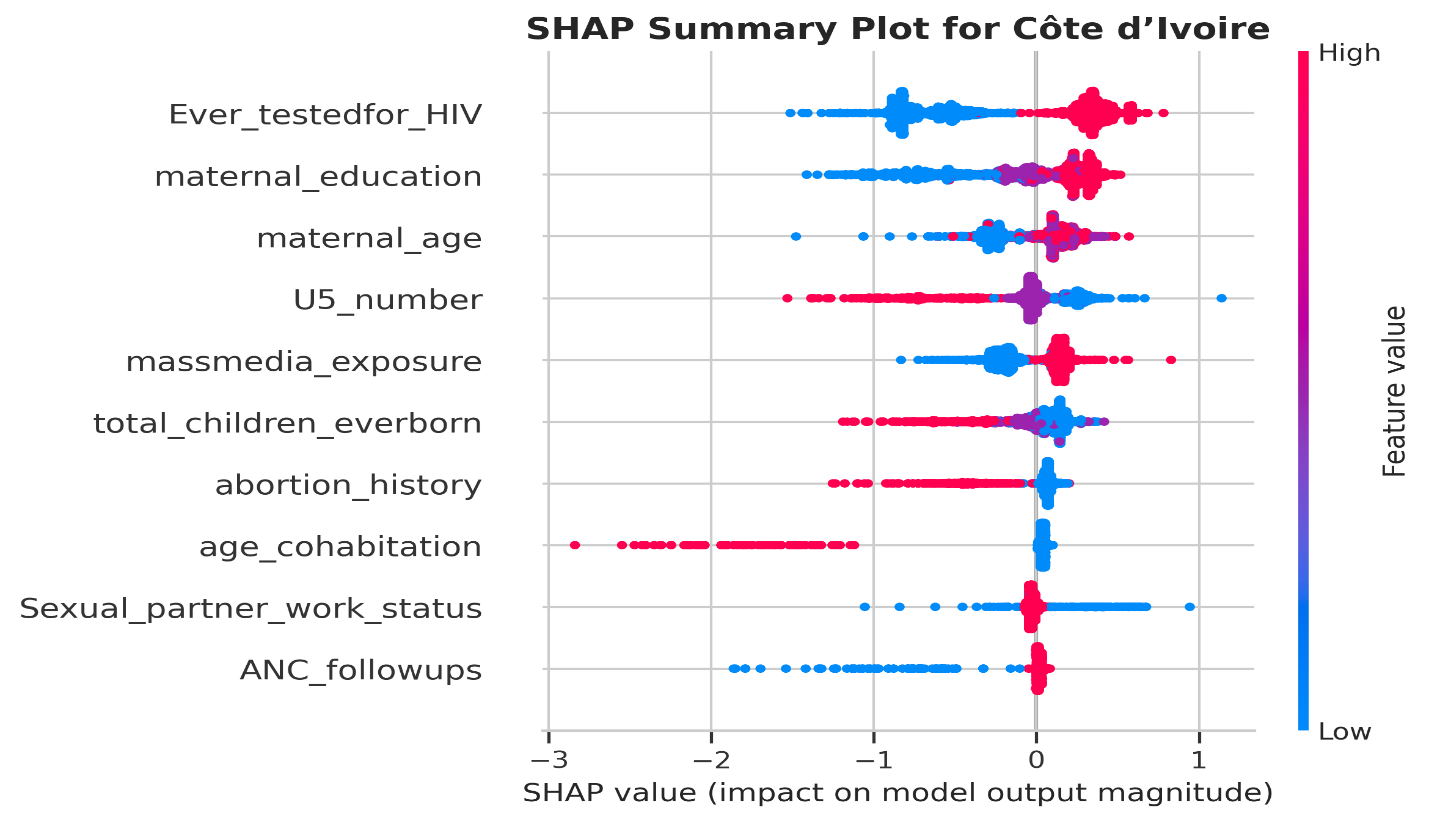


Supplementary Figure 4: SHAP (Shapley Additive exPlanations) summary plot illustrating the impact and direction of each predictor variable on the model’s prediction of women’s awareness and favorable perception of HIV pre-exposure prophylaxis (PrEP) in Côte d’Ivoire.


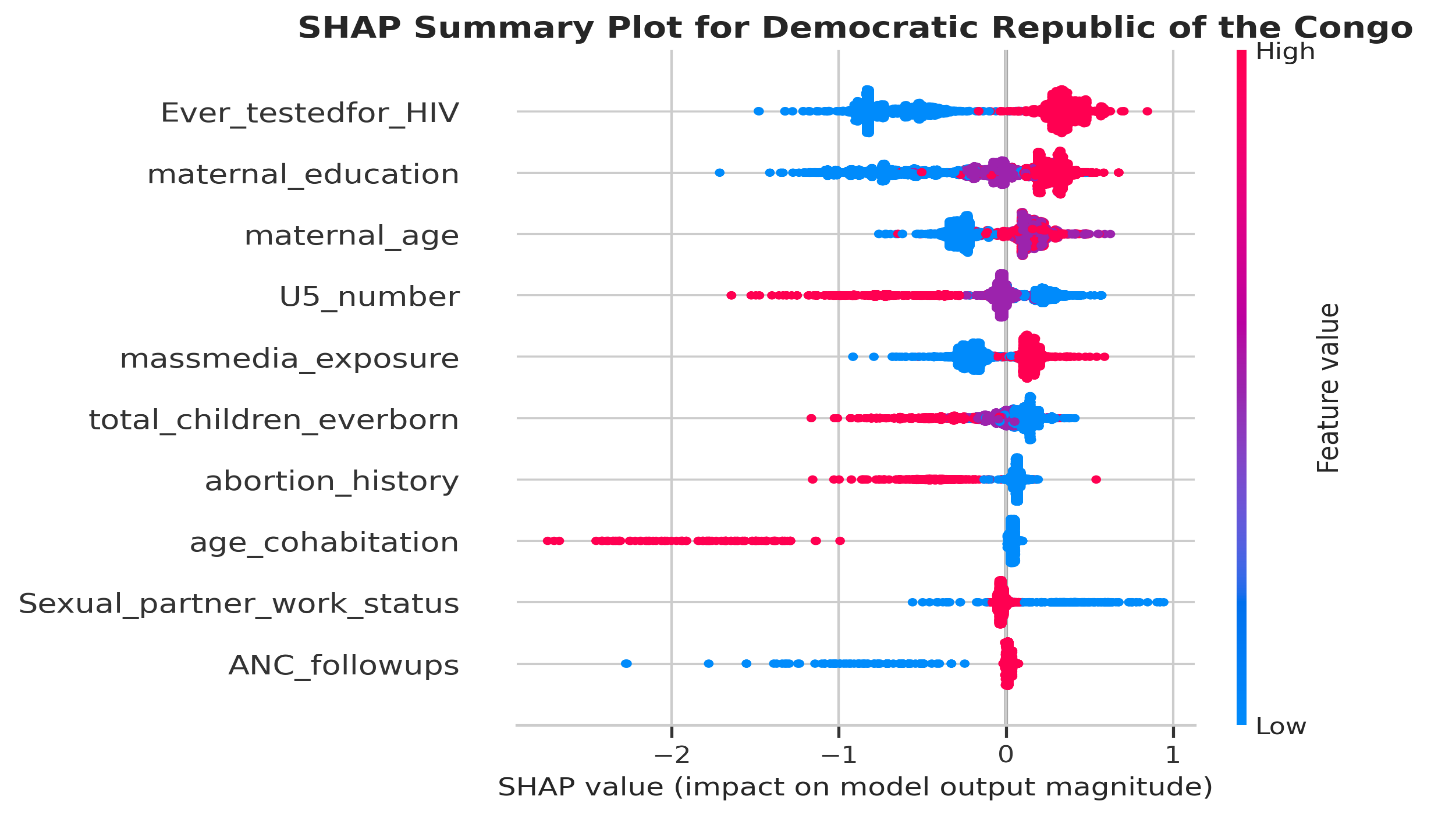


Supplementary Figure 5: SHAP (Shapley Additive exPlanations) summary plot illustrating the impact and direction of each predictor variable on the model’s prediction of women’s awareness and favorable perception of HIV pre-exposure prophylaxis (PrEP) in Democratic Republic of Congo.


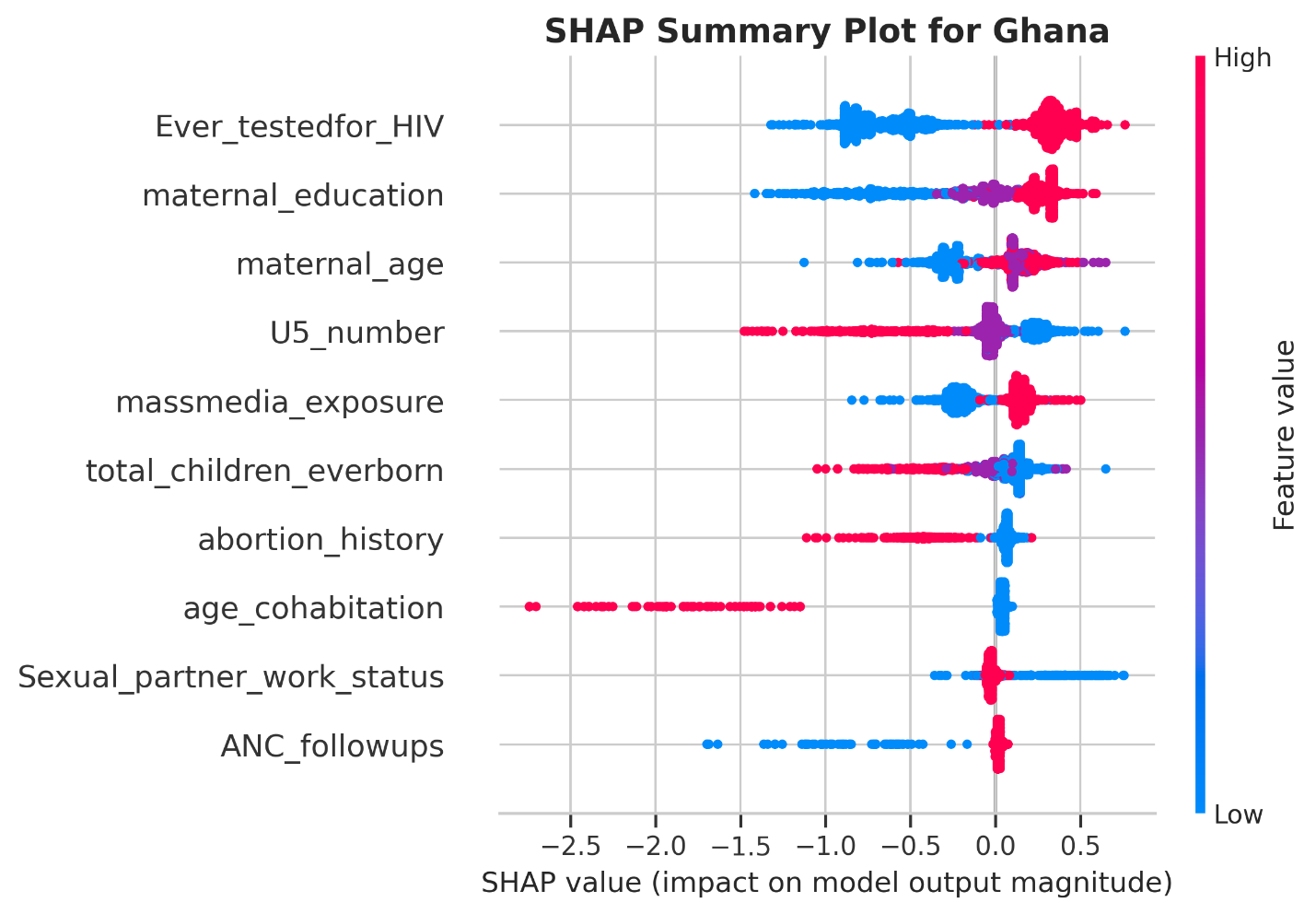


Supplementary Figure 6: SHAP (Shapley Additive exPlanations) summary plot illustrating the impact and direction of each predictor variable on the model’s prediction of women’s awareness and favorable perception of HIV pre-exposure prophylaxis (PrEP) in Ghana.


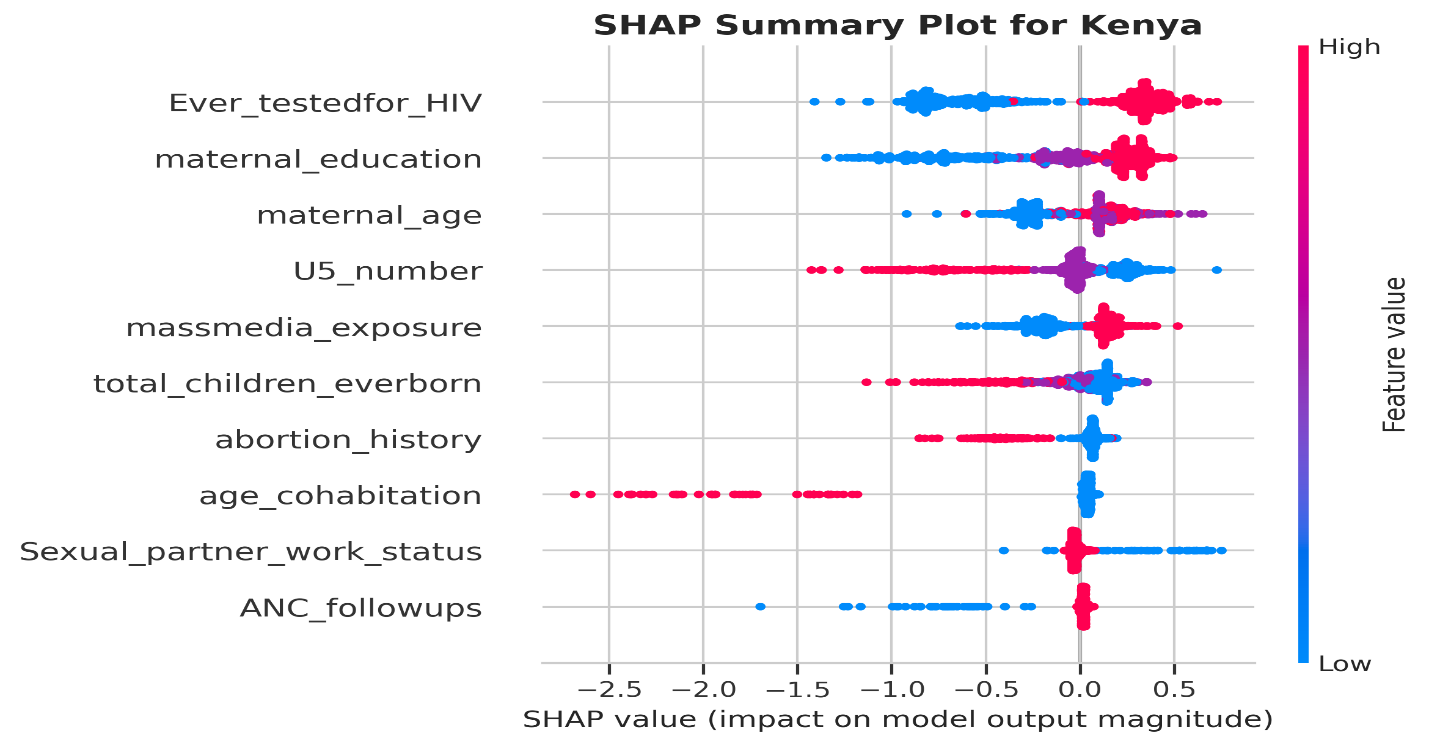


Supplementary Figure 7: SHAP (Shapley Additive exPlanations) summary plot illustrating the impact and direction of each predictor variable on the model’s prediction of women’s awareness and favorable perception of HIV pre-exposure prophylaxis (PrEP) in Kenya.


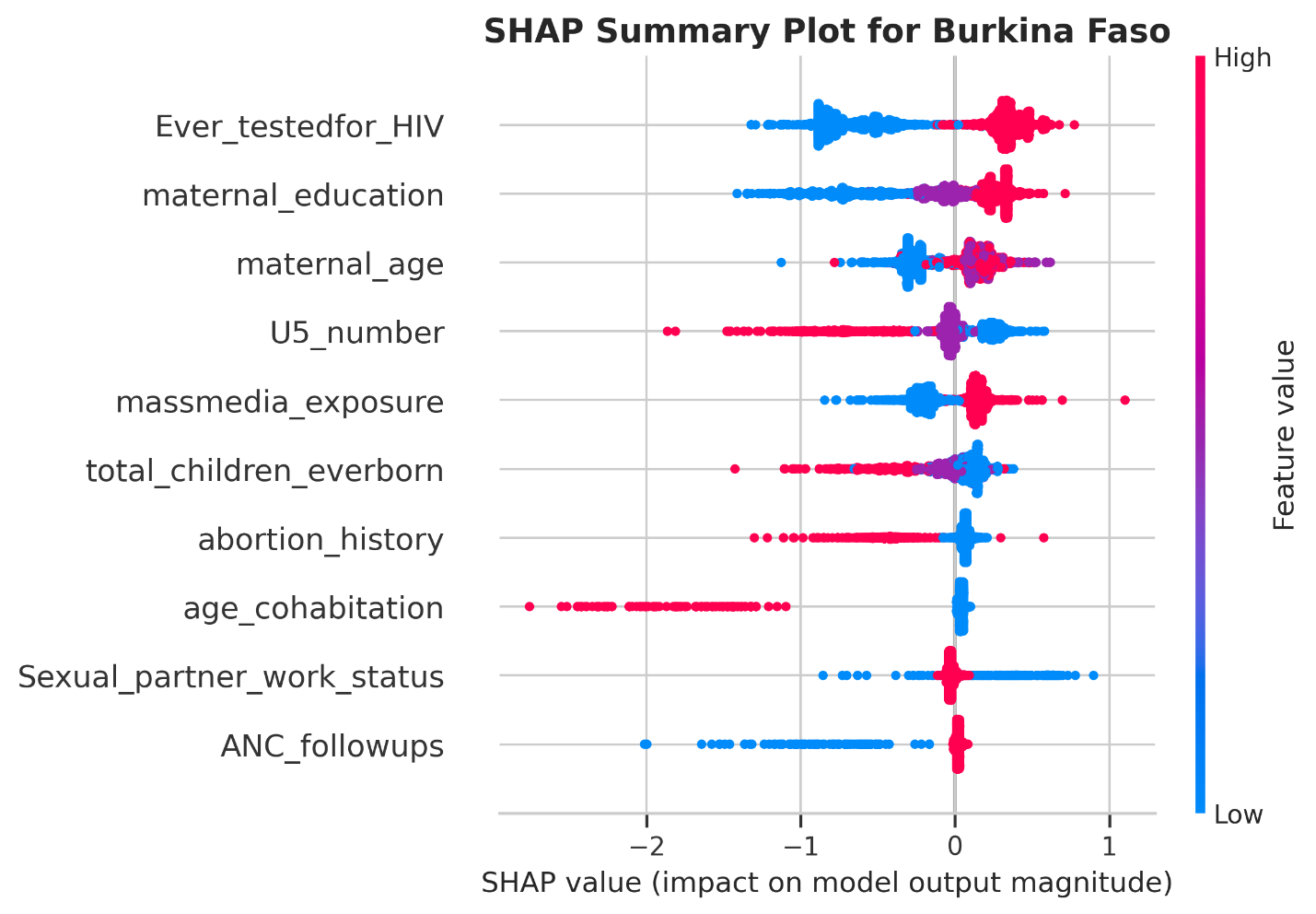


Supplementary Figure 8: SHAP (Shapley Additive exPlanations) summary plot illustrating the impact and direction of each predictor variable on the model’s prediction of women’s awareness and favorable perception of HIV pre-exposure prophylaxis (PrEP) in Burkina Faso.
